# A framework for researching the waterpipe tobacco business sector in the United States

**DOI:** 10.18332/tid/204790

**Published:** 2025-07-23

**Authors:** Seyed Mehrdad Mohammadi, Pamela Ling, Dorie E. Apollonio, Stella Bialous

**Affiliations:** 1 Center for Tobacco Control Research and Education, University of California San Francisco, San Francisco, United States; 2 School of Medicine, University of California San Francisco, San Francisco, United States; 3 School of Pharmacy, University of California San Francisco, San Francisco, United States; 4 School of Nursing, University of California San Francisco, San Francisco, United States

**Keywords:** tobacco industry, waterpipe tobacco, smoking, tobacco products, United States

## Abstract

**INTRODUCTION:**

The waterpipe tobacco sector is a fragmented and multifaceted part of the tobacco industry, including shisha tobacco, hookah devices, and the hospitality industry. Information on this sector's business structure and its actors is limited. This study aims to develop a framework for understanding the scope, business entities, and key actors in the waterpipe sector in the United States that can facilitate research and public health interventions.

**METHODS:**

We first identified a sample of 87 waterpipe-related business entities through internet searches and business intelligence reports provided by marketing research companies. Using grounded theory, we then identified key business components and themes within the waterpipe sector, which we iteratively refined through successive rounds of analysis.

**RESULTS:**

We identified and defined eight domains in the waterpipe tobacco sector: 1) waterpipe tobacco growers, 2) shisha tobacco manufacturers, 3) hookah device and accessories manufacturers, 4) waterpipe charcoal manufacturers, 5) shisha/hookah/charcoal resellers or retailers, 6) waterpipe tobacco service providers and the hospitality sector, 7) waterpipe tobacco networks and trade associations, and 8) aggregated sales. Within these 8 domains, 46 fields of information were identified to construct a detailed information grid for the waterpipe tobacco sector.

**CONCLUSIONS:**

This comprehensive framework and information grid offer a reference base for research, monitoring, and understanding of the waterpipe tobacco sector. Additionally, it can support efforts to improve regulation of the sector in the United States.

## INTRODUCTION

The waterpipe operates by drawing air in through a mouthpiece, which fans and ignites the charcoal placed above a perforated aluminum foil. The burning charcoal, in turn, heats specially prepared flavored tobacco underneath to produce smoke. Subsequently, the air, now mixed with the smoke, is passed through water, generating bubbles, before being inhaled. Shisha, argileh, nargileh, hookah, and qalyan are other words for waterpipe tobacco in different regions of the world and are often used interchangeably^1,2^. Typically, hookah refers to the actual smoking device, and shisha refers to the flavored tobacco consumed^3^. Shisha or mu'assel is tobacco mixed with molasses or honey syrup, humectants (glycerin) and a variety of flavors and aromatic substances^4,5^. The flavoring involves several options, including fruit, mint, chocolate, caramel, beverage-inspired, and blended.

Waterpipe tobacco smoking has become a global public health problem, driven by the introduction of manufactured flavored tobacco, the social dimensions of waterpipe use, dissemination through social media, and gaps in regulatory/policy specific to the waterpipe^6,7^. The flavored smoke of waterpipe and the social dimension all contribute to the consumers’ experience and attraction of the youth^8-10^. Aside from its consumption at homes, waterpipe is typically offered to patrons in specialized venues such as bars, eateries, teashops, or cafes, often in dedicated lounges or open spaces.

In the United States (US), hookah is also associated with a perceived cultural experience and exotic appeal^11,12^. In a 2022 study, the prevalence of current waterpipe tobacco use among middle and high school students in the US was reported at 0.8%^13^. Findings from a 2024 analysis of the Population Assessment of Tobacco and Health (PATH) Study (2013–2021) showed that current waterpipe tobacco use among adults declined from 2.19% in Wave 1 to 1.24% in Wave 6, while ever use increased from 16.39% to 20.92% over the same period^14^. Waterpipe tobacco parlors first opened in the US decades ago in the immigrant quarters of New York and Los Angeles^15,16^. The adoption of waterpipe smoking among young people, was initially reported among college students, in areas surrounding campus and later gaining customer bases in the broader population in the late 20th century^11,15,17^.

The global waterpipe tobacco market was reportedly worth US$ 1.01 billion in 2024, with expected growth, given existing trends, to US$ 1.77 billion by 2031^18^. Over the past few decades, the cigarette industry has been extensively studied by the public health community, increasing knowledge about the size, sales, inner workings, marketing strategies, and lobbying practices of the cigarette industry, both in the US and globally. However, there is limited understanding of the waterpipe tobacco sector of the tobacco industry^10,19,20^, and even less knowledge of emerging trends within the hookah business, such as the integration of cannabis consumption with hookah and the rise of electronic hookah devices.

There have been previous attempts to explore elements within the waterpipe tobacco sector. One research study classified the waterpipe products of exhibitors at an international hookah fair in Germany into seven categories, namely: waterpipe tobacco, waterpipe tobacco substitute, waterpipe apparatus, waterpipe accessories, charcoal, electronic waterpipe, and other products^19^. The authors followed this study with another one^20^ based on interviews with waterpipe companies at a trade exhibition in 2015. They concluded that the waterpipe industry, while fragmented, is gradually evolving into a mature, globalized, and customer-focused sector with connections to the cigarette industry^20^. The 2021 WHO FCTC KH report on the waterpipe tobacco industry found that the main structural feature of the industry is fragmentation, with many small, competing companies and leaders in individual markets, but no monopolies, and the industry is growing. Al-Fakher and Al-Nakhla are likely the two largest waterpipe tobacco companies in the Middle East and North Africa (MENA) region, both having connections to the transnational tobacco industry^10^. A 2024 study of waterpipe tobacco in the US, based on a content analysis of online stores, found that waterpipe tobacco was available in 66 different brands and 1871 unique flavors in the US market^21^. The complexity, fragmentation, and evolution of this sector strongly suggest the need for a comprehensive framework to understand and monitor it, including its potential associations with the broader tobacco industry and its implications for public health in the US. This study aims to develop such a framework to better understand the scope and structure of the waterpipe tobacco sector and to research and monitor its business entities and actors in the US, supporting public health interventions as well.

## METHODS

We developed a business and profiling framework for the waterpipe tobacco sector in the US tobacco industry using an iterative qualitative method that incorporated both inductive and deductive approaches^22^. The method involved three key steps.

### A *Priori* generic framework development

Initially, we established a foundational framework using generic commerce and business entities research and profiling tools, such as Commerce Research Library of US Department of Commerce and Coresignal^23,24^. These profiling tools helped us identify seven primary themes: 1) basic identity data (e.g. name, place and year of establishment); 2) nature of business (e.g. service/manufacturing); 3) strategy and marketing positioning; 4) product/service portfolio; 5) financial and sales performance data; 6) ownership (e.g. for profit/non-profit, private/publicly listed/state-owned); and 7) management (e.g. board of directors and executive leadership).

### Empirical assessment or inductive process

We searched for examples of waterpipe tobacco businesses using Google in November 2023. Our search strategy combined terms like: ‘waterpipe tobacco’, ‘hookah’, ‘shisha’, ‘seller’, and ‘US’. We reviewed 23 pages of search results, encompassing 217 websites, focusing on pages that referenced waterpipe tobacco business entities (e.g. manufacturers, resellers, distributors, or providers) selling in or to the US. One of the authors (SMM) analyzed the ‘Home’, ‘Products’, and ‘About Us’ sections of 64 out of 77 relevant waterpipe-related businesses to identify relevant information themes and compared them to the seven pre-identified themes, noting areas where additional categories or information types were needed. The same review process was applied to the 23 waterpipe tobacco companies listed in business intelligence resources^18,25^. This iterative thematic development was assessed, revised, and approved by other co-authors (PL, DEA, and SB) in regular review meetings. The process was conducted between November 2023 and August 2024. See Supplementary file Section A for the detailed search process and the list of business entities included in our review categorized by their sources (Google search and Business intelligence resources).

### Cross-referencing with WHO FCTC Report and Tobacco Tactics

Finally, in September and October 2024, one author (SMM) reviewed the 2021 report from the WHO Framework Convention on Tobacco Control Knowledge Hub on Waterpipe (WHO FCTC KH)^10^ and the Tobacco Tactics website dedicated page on waterpipe tobacco^26^ to ensure our framework did not miss any relevant business themes or actors of the waterpipe sector identified in those reports. Another author (PL) double-checked this process.

## RESULTS

We use the term ‘waterpipe tobacco sector’ to mean: ‘manufacturers, importers, wholesale distributors, and retailers of waterpipe tobacco, charcoal, and devices as well as waterpipe consumption service providers’. The sector comprises agriculture, manufacturing and service components, with the manufacturing component encompassing the production of shisha tobacco, charcoal, and hookah devices^20,26^. We defined six distinct business identities or classes in this sector as follows and as depicted in Figure 2:

**Figure 1:**
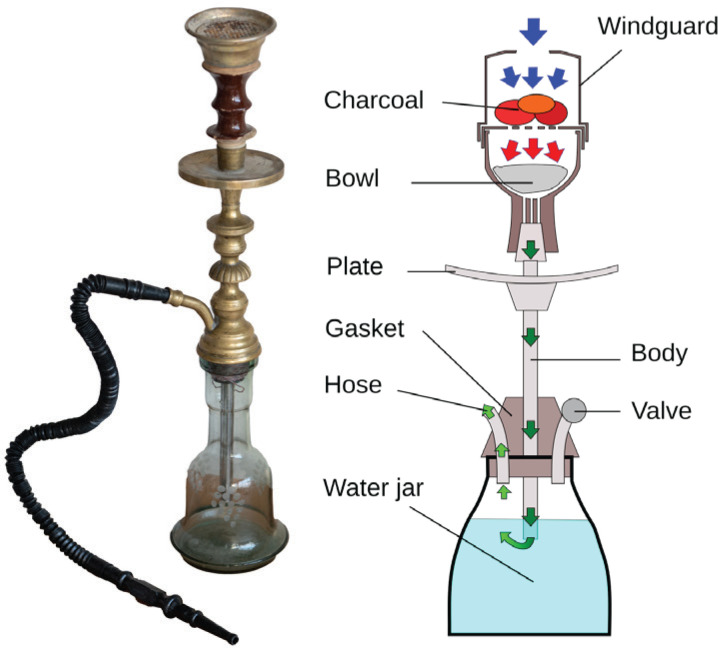
Picture and elements of a waterpipe. The right image is sourced from Wikipedia¹, and the left image is provided courtesy of Aurora Studio, Tehran

**Figure 2:**
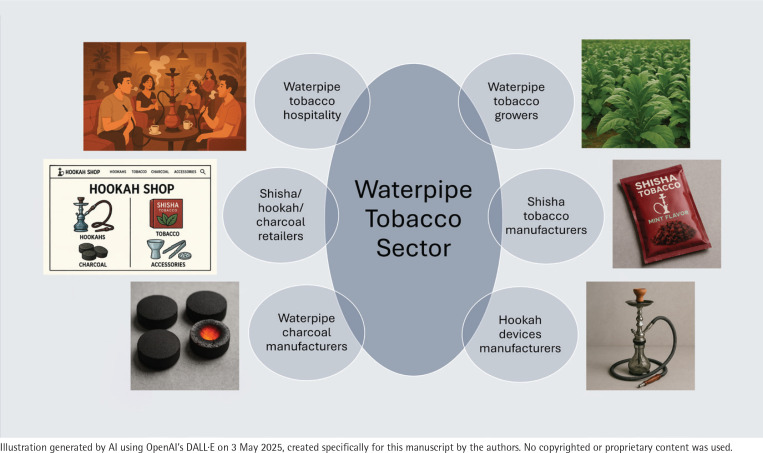
The scope of the waterpipe tobacco sector: Six distinct business entities, based on a qualitative review of 87 waterpipe tobacco related business entities, 2024

1. Waterpipe tobacco grower: a farmer who cultivates tobacco plants to produce tobacco leaves for use in waterpipe tobacco. This is a specialized class of tobacco growers with specific agricultural and curing practices^27^ that are suited for the kind of tobacco characteristics – such as water, sugar and nicotine content – for shisha tobacco manufacturing.
2. Shisha tobacco manufacturer: a company that produces flavored tobacco blends specifically for use in hookahs. This involves processing tobacco leaves and blending them with various flavorings, molasses, and other ingredients. The company may manufacture its own brands, and may have distributors or subsidiaries in countries other than the country of origin.
3. Hookah device and accessories manufacturer: a company that designs, produces, and distributes hookahs – including the base, stem, hose, bowl, and grommets – and accessories like mouthpieces and cleaning tools. We include novel hookah device manufacturers, i.e. a company that creates innovative hookah designs, including innovative materials for body or hose and electric or electronic heaters instead of charcoal that still retain the overall anatomy and mechanism of the traditional hookah apparatus and, at times, may compete with the traditional hookah^20,28^. It is noteworthy that electronic nicotine delivery systems (colloquially called ‘vapes’) carrying shisha or hookah designations such as ‘hookah pens’, ‘shisha pens’, and ‘e-shisha’, of various sizes with little anatomical and/or mechanistic resemblance to traditional hookah are typically categorized as e-cigarettes^10,19,20,28^ and are not included in the framework. Neither does the framework include steam stones, such as shiazo, a novel substitute for hookah tobacco, that produce flavored aerosols when heated for inhalation or for room scent^29^.
4. Waterpipe charcoal manufacturer: a waterpipe charcoal manufacturer is a company that produces charcoal specifically designed for use in hookahs (waterpipes). This involves creating charcoal that lights easily and burns consistently.
5. Shisha, hookah, and charcoal reseller: a business that sells shisha tobacco and hookah products manufactured by another company on a wholesale or retail end-user basis. Resellers typically sell charcoal as well as hookah devices along with shisha tobacco. Retailers can operate through physical stores, online platforms, or a combination of both.
6. Waterpipe tobacco service provider/hospitality: an establishment – in the context of a bar, restaurant, teahouse, hotel, or resort – where customers can smoke flavored tobacco from hookahs. They typically offer a variety of shisha flavors.

Table 1 presents the detailed information grid for the waterpipe tobacco sector under eight domains. The first six domains correspond with the six business identities defined above. We added two additional domains to complete the picture of the waterpipe tobacco business: domain 7 includes aggregated sales information, and domain 8 is information on trade associations, informal organizations, or interest groups in the waterpipe tobacco business that promote their common interests, standards, and cooperation. The detailed list of interest groups in domain 8 can be found in Supplementary file Section B. As previously described, the data type is noted as ‘company/individual’ or ‘aggregated’, for example, the data type for Starbuzz company is ‘company/individual’ and the data type for the State of California annual shisha sales is ‘aggregated’.

**Table 1:** Eight domains and relevant features within the waterpipe tobacco sector based on a qualitative review of 87 waterpipe tobacco-related business entities, 2024

Domain	Data fields
**1. Waterpipe tobacco growers**	Type: Aggregated production (tons/year) 1. Total Broken down by: 2. Imported, domestic 3. Source: US states, countries
**2. Shisha tobacco manufacturers**	Type: Company/Individual Examples: Al-Nakhla, Starbuzz, Flavors of Americas (see the companies’ links in the Supplementary file Section A) Name Country of incorporation Year established Ownership and control Capital (US$) Recent annual sales and income (US$) Market share Key people: founders, directors, president, executives Product catalog: brands, variants, flavors, and non-nicotine/herbal shisha Market positioning: A description of overall strategy, targeted market segments (Black, Latin, Arab, general, etc. populations), focus, unique selling point, pricing, quality, sourcing; company’s story; or characteristic aspect of the company Marketing and public relations: website address, social media presence, blog, and newsletter, sponsorships and communications of scientific nature
**3. Hookah device and accessories manufacturers**	Type: Aggregated Sales volume (in numbers and US$/year) 1. Total Broken down by: 2. Generic, brand 3. Imported, domestic 4. Top 3–5 selling brands 5. Top 3–5 luxury or high-end brands (e.g. above 200 US$ or so per piece) Example: ‘Steamulation’ 6. Top 3–5 Novel brands Example: C2 Hookah (see the company link in the Supplementary file Section A)
**4. Waterpipe charcoal manufacturers**	Type: Company Example: CocoNara (see the company link in the Supplementary file Section A) Data fields the same as shisha tobacco manufacturers
**5. Shisha reseller^a,b^**	Type: Company Example: hookah-shisha.com Name WWW address Country of incorporation Year established Exclusivity: whether the business is dedicated to only selling waterpipe tobacco products or is a diverse product outlet including non-tobacco products (e.g. Amazon) Product catalog: shisha brands and flavors; hookah device and accessories; non-tobacco/herbal shisha Presence of vapes, pods, and liquids co-sale (along with waterpipe products) Wholesale: whether it sells wholesale or only to end-users Marketing: website, social media, etc.
**6. Waterpipe tobacco service provider, establishment or hospitality^c^**	Type: Aggregated Number of establishments (current and trend) 1. Total Broken down by: 2. States/Cities 3. Category of venues: bars, teahouses, cafés, restaurants, and hospitality or tourism resorts 4. Menu: variety of shisha flavors and blends offered 5. Price range
**7. Sales**	Type: Aggregated Shisha tobacco sales and consumption in tons and US$ per year (current and trend) 1. Total Broken down by: 2. States 3. Mode of sale: online outlets and physical shops 4. Demographic breakdown 5. Social characteristics: ethnicity, education, and income 6. Tobacco type: ajami, shisha, nicotine-free shisha, dark leaf, light leaf 7. Hospitality-provided and self-service
**8. Networks, trade associations, or interest groups**	Type: Company/Individual Example: Hookah Chamber of Commerce^d^ 1. Mission 2. Membership 3. Annual budget 4. Source of funding and sponsorship 5. Activities and lobbying data

a Information about shisha, hookah, and charcoal sold by individual internet or physical outlets is overly detailed and outside the scope of our designed framework. However, sales data from shisha and charcoal manufacturers are valuable and included in the Table.

b Similarly, information on brick-and-mortar distributors selling shisha, charcoal, and hookah devices, often alongside other tobacco products, is excluded due to the high number of such outlets and the difficulty in acquiring specialized data. Instead, our focus is on major internet resellers that are identifiable and quantifiable.

c More sophisticated information on the geographical distribution of such establishments is not covered in this information grid.

d List of networks, trade associations, or interest groups (Supplementary file Section B).

## DISCUSSION

Our framework is the first to provide a broad, comprehensive approach to cover and understand the full spectrum of businesses within the waterpipe tobacco sector. It emphasizes the sector’s complexity and fragmentation and by establishing a common language and understanding of the sector’s domains, our framework aims to facilitate future research. While the constructed framework is based on inquiry from US sources and intended for use in research within the US, it may be generalizable and applicable to other countries as well.

The framework highlights significant gaps in knowledge of the sector’s status and business activities. There is little available information on financial and sales data, or insight into the strategic and marketing positioning of shisha tobacco manufacturers. Additionally, information on overall US sales of shisha tobacco, charcoal, and waterpipe services – along with geographical sales data and trends – remains scant. The activities of the trade groups, including their spending and sponsorship, also warrant further investigation.

An important dimension that warrants noting in the US is the role of cultural identity – particularly among migrant communities from regions where waterpipe tobacco use is a long-standing social tradition. These communities may not only constitute a core customer base but could also be over-represented among operators across several of the business identities identified in our analysis. Their cultural practices and geographical clustering may influence both the demand and spatial distribution of waterpipe venues. Recognizing this group as a distinct actor in the waterpipe tobacco landscape is essential, particularly for informing culturally sensitive public health messaging and regulatory approaches. Engaging cultural leaders within these communities may be a necessary step in effective policy development and implementation.

This very culturally distinct presence has often been treated differently – perhaps even more leniently – under public health regulations, particularly with respect to health warnings and flavor bans^26,30,31^. Despite this, there is compelling evidence that supports concerns about waterpipe tobacco use, including its addictiveness, toxicity, potential to serve as a gateway to other nicotine products^32^, and the role of flavors in youth initiation^8-10^. These factors highlight the need for closer scrutiny of the policy activities of waterpipe companies and their allies in the US.

There is limited evidence of transnational tobacco companies’ serious engagement in the waterpipe business over the years. To our knowledge, such involvement has been restricted to Japan Tobacco International’s acquisition of Nakhla in 2013^33^ and Philip Morris’s 21% stock ownership in Godfrey Phillips India Ltd., a major tobacco company in India^34^. Interestingly, the waterpipe sector has adopted electric heating mechanisms from the vaping industry, replacing traditional charcoal while preserving the hookah’s overall design. Given the substantial transformations and acquisitions within the tobacco industry, further developments in this area may occur in the future.

### Limitations

The data sources used in this study are inherently limited. Our analysis is based on a cross-sectional snapshot collected within a constrained time window, relying primarily on publicly available internet searches and website content. We did not have access to proprietary datasets or sales figures. Additionally, the grounded theory approach employed is expert-based and may be subject to interpretive biases and limitations in creative inference.

## CONCLUSIONS

The framework and the information grid for the waterpipe tobacco sector provide a solid reference base for cohesive and complementary research and monitoring of this sector of the tobacco industry in the US. The eight identified and defined domains, along with their sub-themes, are well-suited for systematic descriptive and evaluative research. The framework highlights some key opportunities for research to inform regulation of this sector, including the need for information on US sales of shisha tobacco, charcoal, and waterpipe services along with geographical sales data and trends, and for research on the activities of waterpipe trade groups, including their spending and sponsorship.

## Supplementary Material



## Data Availability

Data sharing is not applicable to this article as no new data were created.
