# Supplementary material for: A framework for researching the waterpipe tobacco business sector in the United States
Source: Tob Induc Dis. 2025 Jul 23;23:10.18332/tid/204790. doi: 10.18332/tid/204790 (PMC12285647; doi:10.18332/tid/204790)
Supplement: Supplementary file 1 [file TID-23-100-s1.pdf]

Supplements to accompany "A Framework for Researching the Waterpipe Tobacco Business Sector in the United States"

Seyed Mehrdad Mohammadi, MD, MPH, MALD*, Pamela Ling, MD, MPH, Dorie E. Apollonio, PhD, MPP, Stella Bialous, DrPH, FAAN*

Center for Tobacco Control Research and Education, University of California San Francisco, San Francisco, CA

* Correspondence: merdad@alumni.tufts.edu, Stella.Bialous@ucsf.edu

Supplement A: Waterpipe tobacco companies or business entities that are included in the review categorized by their sources

Supplement-B: A preliminary list of networks, trade associations, or interest groups

A-Waterpipe tobacco companies or business entities that are included in the review categorized by their sources

1. Google search

We experimented with several keyword combinations of waterpipe tobacco terms, "seller," and "United States" with various results. Ultimately, we used ("waterpipe tobacco" OR hookah OR shisha) AND (seller OR retailer), adding retailer and dropping "United States" since it seemed to have a much better recall without a non-manageable consequence in precision[1,2]. Google algorithm

itself set the area for San Francisco, CA that did not seem to compromise our intended business targets in the United States. The following summarizes the search results, excluded results, and included ones:

- Total pages retrieved: 219 (23 pages of results)
- Excluded pages:
  - Duplicated pages (of the same business entity): 43
  - Non-business pages including states' governments and regulatory agencies, academic, news, Wikipedia, Quora, Reddit, and NGO; online marketplaces; and other tobacco products (vapes, bong, cannabis): 66*
  - Non-US waterpipe pages: 33
- Relevant pages: 77
- Sampled pages: The first 64 pages

* There were 10 seller webpages exclusively focused on vapes, pods, and e-cigarettes—with no connection to classic or novel waterpipe products like e-hookah or e-shisha—that still appeared in the search query. This could either be an issue with Google's algorithm or a result of SEO or meta-data engineering by these websites to increase visibility and attract waterpipe consumers to vapes and pods.

We used the Search Engine Results Page (SERP) information structure to scrape the data[3,4]. Of the Text result, Rich result, Image result, and Video result on SERP, we focused on the Text result and scraped the following four fields:

- Domain: The name or domain of the website
- Title: The clickable title of the page, linking directly to the URL
- URL
- Snippet: The (meta) description summarizing the content of the page

The businesses surveyed are listed below, and snapshots of their homepages can be accessed on eScholarship.org[5].

	Domain	URL
1.	5starhookah	https://5starhookah.com/
2.	Alibaba	https://www.alibaba.com/showroom/best-seller-shisha.html
3.	Aliexpress	https://www.aliexpress.us/item/3256803828416696.html?gatewayAdapt=glo2usa4itemAdapt

4. Amazon https://www.amazon.com/Hookahs/b?ie=UTF8&node=10342619011
5. Arabian Hookah Lounge http://arabianhookahlounge.com/store.html
6. Black Shisha https://blackshisha.com/
7. Blakk Smoke https://www.blakksmoke.com/hookah-pens
8. Chargeitshisha https://chargeitshisha.com/
9. Chicha Shack http://www.chichashack.com/
10. Cocoyaya https://cocoyaya.in/
11. Connis Hookah https://connishookah.com/
12. Ebay https://www.ebay.com/sch/112485/i.html?_from=R40&_nkw=hookah
13. El-Badia https://www.el-badia.com/en/
14. Fumari https://www.fumari.com/
15. Guess Hookah https://guesshookah.com/
16. Headed West https://theheadedwest.com/products/denver-hookahs-and-shisha-tobacco/
17. Hi-techclub https://hi-techclub.com/en/
18. Holy Smoke Shisha Bar https://holysmokeshishabar.com.cy/
19. Hookah Canada https://www.myhookah.ca/
20. Hookah Guy https://www.hookahguy.com/
21. Hookah John https://hookahjohn.com/
22. Hookah Market https://hookahmarket.com/
23. Hookah Merch https://www.hookahmerch.com/
24. Hookah Paradise https://hookahparadise.com/
25. Hookah Set https://www.hookahset.com/
26. Hookah Shisha https://www.hookah-shisha.com/
27. Hookah Vault https://www.hookahvault.com/
28. Hookah Wholesalers https://www.hookahwholesalers.com/
29. Hookah Zone https://shophookahzone.com/
30. Hydro Brand https://www.hydrobrand.us/c-21-hydro-nicotine-free-shisha-50g.aspx
31. Icon Hookah https://iconhookah.com/
32. Kaloud https://kaloud.com/
33. Kaya Shisha https://www.kaya-shisha.com/
34. Khalil Mamoon https://khalilmamoon.com/collections/hookahs

35.	Medwakh	https://www.medwakh.com/
36.	Mob Hookah	https://www.mobhookah.com/
37.	My Shopify	https://theme204-hookah.myshopify.com/
38.	Mya Hookah	https://myahookah.com/
39.	Oxide Hookah	https://www.oxidehookah.com/
40.	Pharaohs Hookahs	https://pharaohshookahs.com/collections/all-hookahs
41.	Premium Hookahs	https://premium-hookahs.com/
42.	Shisha Bucks USA	https://www.shishabucks.com/shop/
43.	Shisha Depot	https://shishadepot.ca/
44.	Shisha Gear	https://shishagear.com/
45.	Shisha Mart	https://shishamart.ca/
46.	Shisha Original	https://shishaoriginal.com/en/
47.	Shisha Steamulation	https://shisha-steamulation.de/en/store/
48.	Shop Overdozz	https://shopoverdozz.com/collections/shisha
49.	Shop Starbuzz	https://shopstarbuzz.com/
50.	Shopdop	https://shopdop.in/
51.	Smoke Shop in Dallas	https://smokeshopindallas.com/hookah-shisha-store-plano-tx/
52.	South Smoke	https://www.southsmoke.com/
53.	Texas Hookah	https://www.texashookah.com/
54.	Texas Hookah Premium	https://www.texashookahpremium.com/
55.	The Hookah Lab	https://thehookahlab.com/
56.	The Hookah Shop	https://thehookahshop.com/
57.	The Shisha Shop	https://www.theshishashop.com/
58.	Tobaccos of Hawaii	https://www.tobaccosofhawaii.com/content/hookahs-and-shisha
59.	UENI Website	https://hook-it-up-hookah.ueniweb.com/products/smoking/elite-rising-hookah-50649536
60.	Vapospy	https://www.vapospy.com/p/stundenglass-gravity-hookah
61.	World Hookah Market	https://worldhookahmarket.com/
62.	World of Shisha	https://www.worldofshisha.com.au/
63.	X-hale Hookah	https://www.hookahomaha.com/online-store
64.	Zahrah USA	https://zahrahusa.com/

2. Business intelligence resources

Based on various business intelligence resources, we studied the websites of the following companies, and snapshots of their homepages are available on eScholarship.org[5].

	Company	URL
1.	Al Fakher	https://alfakher.com/en-us/
2.	Al Rayan Hookah Tobacco	https://www.alrayanmolasses.com/en/Home
3.	Al-Amir Tobacco	https://alamirtobacco.com/
4.	Alchemist Tobacco	https://www.instagram.com/alchemisttobacco/
5.	Altawareg Shisha Tobacco	https://www.instagram.com/altawaregshisha/
6.	Alwaha-Tobacco	https://alwaha-tobacco.com/
7.	Cloud Tobacco	http://www.cloudtobacco.com/
8.	Dekang	https://www.dekangbio.com/
9.	Eastern Company*	https://www.easternegypt.com/?lang=en
10.	Fantasia Company	https://www.fantasiacompany.com/
11.	Flavors of Americas	https://www.foa.com.py/en/
12.	Fumari	https://www.fumari.com/
13.	Godfrey Phillips India*	https://www.godfreyphillips.co.in/
14.	Haze Tobacco	https://hazetobacco.com/
15.	Mazaya	https://officialmazayafamily.com/ (USA distributor)
16.	Mujeeb Sons	https://mujeebsons.com/index.php
17.	Nakhla Tobacco (Japan Tobacco Inc.**)	https://nakhla.com/
18.	Prince Molasses	http://www.prince-molasses.com/web/index.aspx
19.	Romman Shisha	https://rommanshisha.weebly.com/
20.	Social Smoke	https://www.socialsmoke.com/
21.	SOEX	https://soex.com/
22.	Starbuzz Tobacco	https://starbuzztobacco.com/
23.	Ugly Hookah	https://uglyhookah.com/

* According to Research and Markets business intelligence source, both Philip Morris International (PMI) and British American Tobacco (BAT) are active in the waterpipe tobacco

market[6]. Additionally, according to Data Bridge Market Research, Godfrey Phillips India Ltd (GPI) is involved in the shisha tobacco business[7]. We also know that PMI owns minority stakes in GPI[8]. GPI's product portfolio only includes cigarettes, leaf and cut tobacco, and confectionery, without mentioning shisha or waterpipe tobacco[9]. Eastern Tobacco Company produces waterpipe tobacco and common brands of transnational tobacco companies[10].

** In 2013, Japan Tobacco Inc. acquired Nakhla Tobacco Company, a major waterpipe tobacco manufacturer present in Egypt and exports to 85 countries, primarily in the Middle East and North Africa[11,12].

B-A preliminary list of networks, trade associations, or interest groups

These organizations can be the subject of further research.

1. National Hookah Community Association
   https://www.nationalhookah.com/
2. European Shisha Community Alliance
   https://shishaalliance.org/
3. Hookah Chamber of Commerce
   https://www.hookahchamber.com/
   https://www.facebook.com/hookahchamber
4. Hookah University
   https://www.hookah-university.com/
5. Hookah / Shisha Guides and Reviews
   https://www.hookah.org/
6. World Shisha Dubai
   https://worldshishaevents.com/
7. Hookah Battle
   https://hookahbattle.com/en/
   https://www.youtube.com/@hookahbattle
8. Shisha Network
   https://shisha.network/

9. Hookah Lounge Directory - Find Hookah Lounges - Trusted Hookah Lounge Reviews Formerly, Hookah Exclusive Magazine https://www.hookahlounges.org/
10. Shisha and Hookah Community iOS app https://apps.apple.com/us/app/shisha-and-hookah-community/id1608081348

References

1. Mamillapally N, Mulukutla T. An analytical study of meta search engines performance based on precision and relative recall. International Journal of Electronics Communication and Computer Engineering. 2013;4(6):144-149.
2. Precision, Recall and F-Measure of Select Search Engines: Retrieval of Research Article in the Field of Library and Information Science - ProQuest. Accessed August 12, 2024. https://www.proquest.com/openview/95541f515f0599dfb6b40c95a85b777d/1?pq-origsite=gscholar&cbl=5318417
3. Snap Agency. Anatomy of a Google Search Engine Results Page (SERP) in 2023. Published January 12, 2023. Accessed August 12, 2024. https://web.archive.org/web/20240812221902/https://www.snapagency.com/anatomy-of-a-google-search-engine-results-page-serp/
4. Visual Elements Gallery of Google Search | Google Search Central | Documentation | Google for Developers. Accessed August 12, 2024. https://web.archive.org/web/20240810172419/https://developers.google.com/search/docs/appearance/visual-elements-gallery
5. Mohammadi SM, Ling P, Apollonio DE, & Bialous S. (2024). Appendix: Websites Homepages Snapshots to accompany “A Framework for Researching the Waterpipe Tobacco Business Sector in the United States.” UCSF: Center for Tobacco Control Research and Education. Retrieved from https://escholarship.org/uc/item/4r80b5pd
6. Hookah Tobacco Market 2024-2028 - Research and Markets. Accessed September 2, 2024. https://web.archive.org/web/20240902102757/https://www.researchandmarkets.com/report/hookah-tobacco#tag-pos-2

7. Shisha Tobacco Market Forecast, Share, Strategies, Scope, Overview | Data Bridge Market Research. Accessed September 2, 2024. https://web.archive.org/web/20240902104009/https://www.databridgemarketresearch.com/reports/global-shisha-tobacco-market
8. "Godfrey Phillips India." Wikipedia, The Free Encyclopedia. Accessed July 22, 2024.https://en.wikipedia.org/w/index.php?title=Godfrey_Phillips_India&oldid=1216805298
9. Godfrey Phillips India Ltd. Accessed September 2, 2024. https://web.archive.org/web/20240901052711/https://www.godfreyphillips.co.in/
10. Towards understanding the Waterpipe Industry in the Middle East | WHO FCTC. Published April 7, 2021. Accessed July 24, 2024. https://extranet.who.int/fctcapps/fctcapps/fctc/kh/wts/news/towards-understanding-waterpipe-industry-middle-east
11. JTI completes acquisition of a leading waterpipe tobacco (Shisha) company. Accessed July 22, 2024. https://web.archive.org/web/20240411103821/https://www.jti.com/news-views/newsroom/jti-completes-acquisition-leading-waterpipe-tobacco-shisha-company
12. Leading Hookah Tobacco Brands - Verified Market Research. Accessed July 22, 2024. https://web.archive.org/web/20230129083201/https://www.verifiedmarketresearch.com/blog/leading-hookah-tobacco-brands/

© 2025 Mohammadi S.M. et al.
